# Endopharyngeal Ultrasound-Guided Transpharyngeal Needle Aspiration for Confirming Retropharyngeal Lymph Nodes’ Metastasis among Nasopharyngeal Carcinoma Patients

**DOI:** 10.34133/cancomm.0004

**Published:** 2026-01-23

**Authors:** Chuanbo Xie, Long-Jun He, Wencheng Tan, Jindong Xie, Yin Li, Lizhi Liu, Guangyu Luo, Kunhao Bai, Hai-Qiang Mai, Guokai Feng, Jun Ma, Jian-Jun Li

**Affiliations:** ^1^ Cancer Prevention Center, State Key Laboratory of Oncology in South China, Guangdong Key Laboratory of Nasopharyngeal Carcinoma Diagnosis and Therapy, Guangdong Provincial Clinical Research Center for Cancer, Sun Yat-sen University Cancer Center, Guangzhou, Guangdong, P. R. China.; ^2^ Department of Endoscopy, State Key Laboratory of Oncology in South China, Guangdong Key Laboratory of Nasopharyngeal Carcinoma Diagnosis and Therapy, Guangdong Provincial Clinical Research Center for Cancer, Sun Yat-sen University Cancer Center, Guangzhou, Guangdong, P. R. China.; ^3^ Department of Oncology, State Key Laboratory of Oncology in South China, Guangdong Key Laboratory of Nasopharyngeal Carcinoma Diagnosis and Therapy, Guangdong Provincial Clinical Research Center for Cancer, Sun Yat-sen University Cancer Center, Guangzhou, Guangdong, P. R. China.; ^4^ Department of Medical Imaging Center, State Key Laboratory of Oncology in South China, Guangdong Key Laboratory of Nasopharyngeal Carcinoma Diagnosis and Therapy, Guangdong Provincial Clinical Research Center for Cancer, Sun Yat-sen University Cancer Center, Guangzhou, Guangdong, P. R. China.; ^5^ Department of Nasopharyngeal Carcinoma, State Key Laboratory of Oncology in South China, Guangdong Key Laboratory of Nasopharyngeal Carcinoma Diagnosis and Therapy, Guangdong Provincial Clinical Research Center for Cancer, Sun Yat-sen University Cancer Center, Guangzhou, Guangdong, P. R. China.; ^6^ Department of Radiation Oncology, State Key Laboratory of Oncology in South China, Guangdong Key Laboratory of Nasopharyngeal Carcinoma Diagnosis and Therapy, Guangdong Provincial Clinical Research Center for Cancer, Sun Yat-sen University Cancer Center, Guangzhou, Guangdong, P. R. China.

Nasopharyngeal carcinoma (NPC) is a malignant epithelial carcinoma located in the head and neck region, with a particularly high prevalence in southern China and Southeast Asia [1]. Radiotherapy is the primary treatment for nondisseminated NPC, yielding a 5-year overall survival rate exceeding 80% [2]. However, a certain proportion of NPC patients develop enlarged retropharyngeal lymph nodes (RLNs) after radiotherapy [3]. Accurately determining whether these enlarged RLNs represent a recurrence of NPC is essential for informing clinical decision-making [4]. Magnetic resonance imaging (MRI) is the mainstay for diagnosing RLN metastasis due to superior soft tissue resolution [5]. A previous study demonstrated that the minimal axial diameter, the status of central necrosis (present or absent), groups of 2 or more RLNs, or any medial RLNs were the main characteristics of metastatic or recurrent RLNs [6]. Nonetheless, enlarged RLNs are not invariably indicative of metastasis, and conversely, smaller RLNs may already harbor metastatic cancer cells [7]. Therefore, reliance on radiological criteria to determine whether the enlarged RLNs are NPC recurrences might lead to overtreatment or missed diagnoses.

To address this limitation, we developed endopharyngeal ultrasound-guided transpharyngeal needle aspiration (EPUS-TPNA), a minimally invasive technique (Fig. 1), through which RLN tissue samples were obtained for further pathological and cytological examinations (Fig. 1). In this retrospective study, we aimed to compare the accuracy of EPUS-TPNA and MRI in diagnosing RLN recurrence based on a relatively long observation period and develop an EPUS-TPNA-based clinical decision system for NPC patients with enlarged RLN after radiotherapy. The detailed protocol for this study is provided in the Supplementary Materials. A total of 149 NPC patients were included in the analyses, of which 72 (72/149; 48.3%) were from the EPUS-TPNA group, while 77 (77/149; 51.7%) were from the MRI-only group (Table S1). The clinical characteristics, including T stage (*P* = 0.563), N stage (*P* = 0.883), and RLN size (*P* = 0.420) at initial diagnosis, were comparable between the EPUS-TPNA group and the MRI-only group, and the key findings are summarized below.

**Fig. 1.**
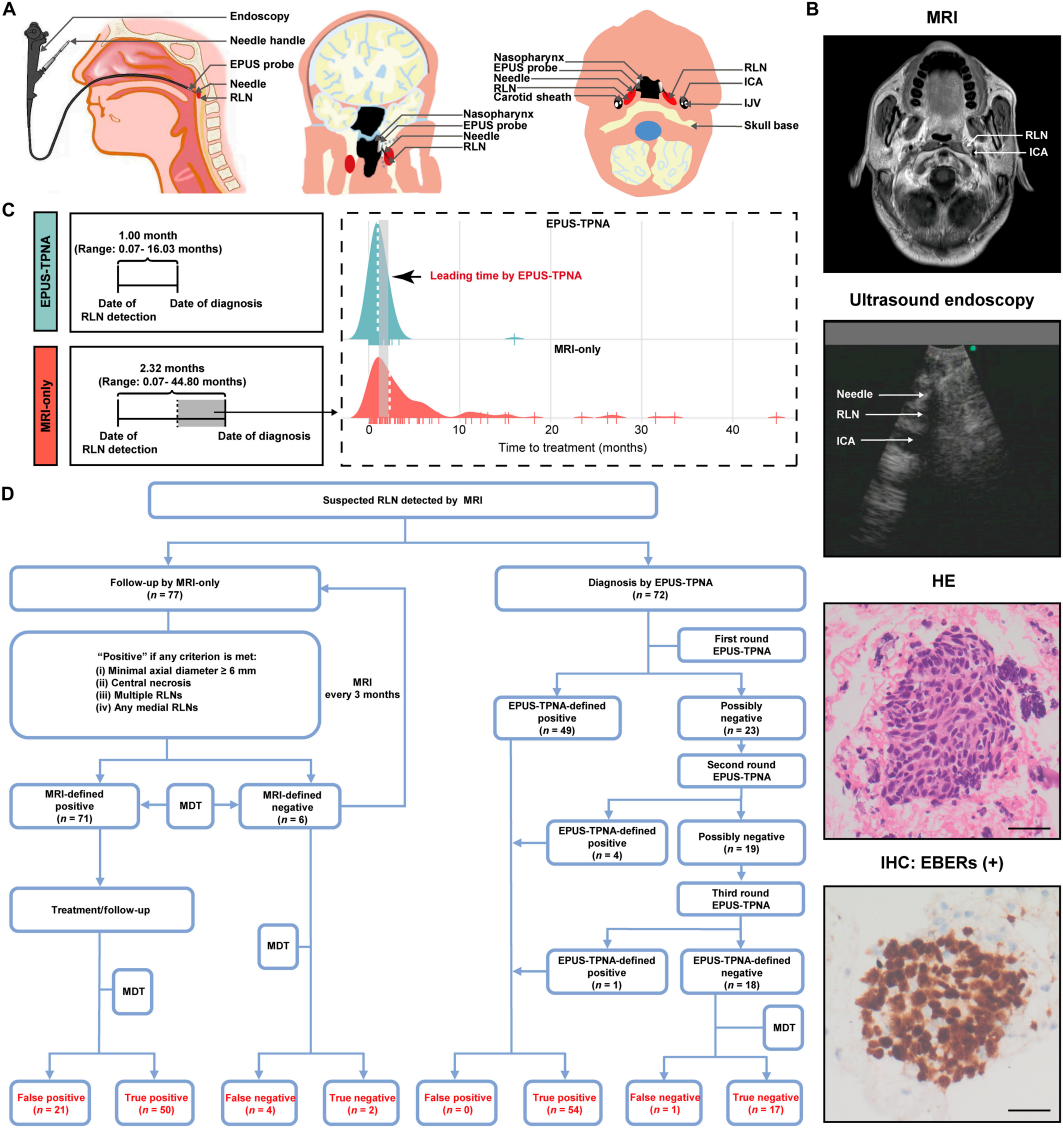
The early diagnostic value of endopharyngeal ultrasound-guided transpharyngeal needle aspiration (EPUS-TPNA) in retropharyngeal lymph nodes (RLNs): Comparison with magnetic resonance imaging (MRI) and clinical workflow. (A) Sagittal plane showing the RLN positioned at the posterior pharyngeal wall, indicating that it could be accessed endoscopically through the nasopharyngeal cavity. The coronal plane depicts the shortest pathway for EPUS-TPNA to reach the RLN. The transverse plane reveals that EPUS-TPNA aspirated the RLN tissue through the nasopharyngeal cavity, thereby avoiding injury to the carotid sheath (from left to right). (B) Representative images [MRI, ultrasound endoscopy, hematoxylin-eosin (HE) staining, and immunohistochemistry (IHC) staining] showing the tissues obtained using the EPUS-TPNA technique have been pathologically confirmed to be NPC (from top to bottom). (C) Schematic representation of several important time points during the treatment of NPC patients with enlarged RLN, and ridge plots showing the leading time by EPUS-TPNA. (D) Clinical routines of EPUS-TPNA-guided and MRI-guided treatments for NPC patients with enlarged RLN after radiotherapy. Patients in the EPUS-TPNA group received a first round of EPUS-TPNA examination. Then, those with negative pathology but still suspected of the possibility of NPC recurrence were recommended for a second and third round of EPUS-TPNA. After 3 rounds of EPUS-TPNA examinations, the negative cases were followed up by MRI to confirm whether the RLNs were metastatic. The enlarged RLN of patients in the MRI-only group was defined as positive if the minimal RLN axial diameter with a minimal axial diameter of 6 mm or larger exhibited central necrosis, presence of multiple RLNs, or medial RLNs. Positive cases were recommended for salvage treatments, while negative cases were followed up by MRI every 3 months to confirm the presence or absence of NPC recurrence in the RLNs. ICA, internal carotid artery; IJV, internal jugular vein; EBERs, Epstein–Barr virus-encoded small RNAs; MDT, multi-disciplinary team.

First, EPUS-TPNA markedly shortened the mean time to diagnose NPC recurrence (Fig. 1). Compared with MRI, EPUS-TPNA could substantially reduce the mean time interval (1.00 month, range = 0.07 to 16.03 months for EPUS-TPNA group versus 2.32 months, range = 0.07 to 44.80 months for MRI-only group) from the date of detecting RLN to the date of confirming the status of the enlarged RLN, enabling faster initiation of appropriate treatment. Second, EPUS-TPNA outperformed MRI in key diagnostic metrics. Fig. 1 shows the results of the EPUS-TPNA examination after each round of EPUS-TPNA. In the MRI-only group, 71 of 77 patients were considered as having metastatic RLNs according to the standard criteria. However, after a mean follow-up of 7.9 months, 21 of these 71 NPC patients initially identified as positive were considered false positives because the RLNs remained stable on MRI (patients who underwent nonsurgical treatment) or were pathologically negative (patients who underwent surgery). Of the 6 patients for whom MRI suggested no recurrent RLN metastasis, 2 were confirmed as true negatives (lesion remained stable or decreased in size), while the remaining 4 were false negatives because the lesion increased in size according to the Response Evaluation Criteria in Solid Tumors (RECIST) 1.1 criteria (Table S2). Compared with the MRI-only group, the EPUS-TPNA group had significantly higher accuracy in diagnosing NPC recurrence in RLNs (98.6% versus 67.5%, *P* < 0.001). Besides, the sensitivity (0.982 versus 0.926), specificity (1.000 versus 0.087), positive predictive value (PPV; 1.000 versus 0.704), and negative predictive value (NPV; 0.944 versus 0.333) of the EPUS-TPNA group were also higher than those of the MRI-only group. Over 90% (i.e., 91.3%, *n* = 21) of true negative patients would be misclassified as NPC recurrence by MRI, compared with none in the EPUS-TPNA group. In addition, 7.4% (*n* = 4) of the true positive patients were misclassified as negative by MRI, while only 1.8% (*n* = 1) of true positive patients were misclassified as negative by EPUS-TPNA.

Accumulating evidence has suggested that RLNs have significant prognostic value for estimating the long-term survival of these patients [8,9]. Currently, the diagnosis of RLN metastasis relies primarily on imaging examinations due to its deep anatomical location. Thus, the National Comprehensive Cancer Network guidelines recommend that these patients should be followed up for several months to confirm the metastatic potential of their enlarged RLN. However, the imaging criteria currently in use are not sufficiently accurate to determine the true status of enlarged LNs. This study identified marked limitations of MRI in determining the metastatic status of enlarged RLNs. Reliance on radiologic examinations alone to guide clinical treatment may expose a substantial proportion of patients to overtreatment, which can lead to unnecessary yet preventable treatment-related adverse events, impaired quality of life, increased financial burden, and even treatment delay with subsequent negative impacts on survival outcomes. Additionally, although pathological examination is the gold standard, existing techniques like computed tomography (CT)-guided mandibular puncture have several limitations, such as long needle path, non-real-time guidance, and small sample size [10].

Our EPUS-TPNA approach is performed under real-time ultrasound guidance, with needle access to the retropharyngeal space via the nostril and avoidance of passage adjacent to delicate vital structures including the carotid sheath and cranial nerves—supporting a superior safety profile for EPUS-TPNA over CT-guided aspiration. Besides, EPUS-TPNA can also be widely used for confirming the RLN status of patients with other head and neck cancers or diseases. For instance, a schwannoma was diagnosed in one patient who had an enlarged RLN with persisting headaches for half a year using this technique. Thus, the clinical prospects of EPUS-TPNA seem promising in broader settings. While positron emission tomography (PET)-CT might demonstrate greater accuracy than MRI in diagnosing lymph node metastasis, its routine use is limited by its cost, associated radiation risks, and the observation that several NPC patients exhibit atypical PET-CT signals following radiotherapy.

Despite these promising results, our study was subject to several limitations. First, due to unavailable nasopharyngeal-specific endoscopes, we used a small-sized ultrasound bronchoscope, which presented challenges in navigating the nasopharyngeal passage and increased the risk of injury to the adjacent mucosa and blood vessels, although no injuries occurred. Second, despite being a minimally invasive examination, EPUS-TPNA might not be applicable for specific patients who are listed in the exclusion criteria, and we intend to design an alternative probe for obtaining these RLN tissues. Third, the limited quantity of RLN tissue acquired through a single EPUS-TPNA procedure may necessitate multiple aspirations, potentially inducing some discomfort for patients. Nonetheless, post-procedure feedback indicated that all patients found the procedure tolerable. Fourth, the metastatic rate among enrolled participants was high (109/149, 73.2%), which may be related to the retrospective nature of this study and could have somewhat affected the results, although it likely did not affect the overall outcomes.

In summary, EPUS-TPNA proved to be a simple, safe, and effective method to obtain RLN tissues for pathological examination. It positively impacted clinical treatment decision-making and markedly reduced the risk of overtreatment compared with standard MRI; thus, EPUS-TPNA has the potential to replace traditional radiologic criteria in diagnosing NPC metastasis. However, a longer follow-up is still needed to validate these observations. Moreover, the application of adjunctive molecular diagnostic methods (such as reverse transcription–quantitative polymerase chain reaction for known biomarkers of NPC) to analyze the needle biopsy samples might help to reduce the demand to aspirate a large volume of tissue samples or even reduce repeat sampling, thereby minimizing harm to the patients.

## Ethical Approval

This study was approved by the Institutional Review Board of Sun Yat-sen University Cancer Center (approval number: XJS2016-016-01) and was registered at ClinicalTrials.gov (identifier NCT03006588). This study was in accordance with the Declaration of Helsinki. Written informed consent was obtained from all participants.

## Data Availability

The authenticity of this manuscript has been validated by uploading the key raw data to the Research Data Deposit public platform (www.researchdata.org.cn) under approval RDD number RDDA2025255453. All data generated or analyzed during this study are included in this published article and its supplementary information files or from the corresponding author upon reasonable request.
