# Supplementary material for: Endopharyngeal Ultrasound-Guided Transpharyngeal Needle Aspiration for Confirming Retropharyngeal Lymph Nodes’ Metastasis among Nasopharyngeal Carcinoma Patients
Source: Cancer Commun (Lond). 2026 Jan 23;46:0004. doi: 10.34133/cancomm.0004 (PMC12857757; doi:10.34133/cancomm.0004)
Supplement: Supplementary 1 — Materials and Methods Tables S1 and S2 Movie S1 [file cancomm.0004.f1.zip › 00079-Supplementary Materials-final.docx]

**Supplementary Materials**

**Endopharyngeal ultrasound-guided transpharyngeal needle aspiration for confirming retropharyngeal lymph nodes’ metastasis among nasopharyngeal carcinoma patients**

Chuanbo Xie^1,†^, Long-Jun He^2,†^, Wencheng Tan^2,†^, Jindong Xie^3,†^, Yin Li^2,†^, Lizhi Liu^4^, Guangyu Luo^2^, Kunhao Bai^2^, Hai-Qiang Mai^5^, Guokai Feng^3,*^, Jun Ma^6,*^, Jian-Jun Li^2,*^

1. Cancer Prevention Center, State Key Laboratory of Oncology in South China, Guangdong Key Laboratory of Nasopharyngeal Carcinoma Diagnosis and Therapy, Guangdong Provincial Clinical Research Center for Cancer, Sun Yat-sen University Cancer Center, Guangzhou, Guangdong, P. R. China;
2. Department of Endoscopy, State Key Laboratory of Oncology in South China, Guangdong Key Laboratory of Nasopharyngeal Carcinoma Diagnosis and Therapy, Guangdong Provincial Clinical Research Center for Cancer, Sun Yat-sen University Cancer Center, Guangzhou, Guangdong, P. R. China;
3. Department of Oncology, State Key Laboratory of Oncology in South China, Guangdong Key Laboratory of Nasopharyngeal Carcinoma Diagnosis and Therapy, Guangdong Provincial Clinical Research Center for Cancer, Sun Yat-sen University Cancer Center, Guangzhou, Guangdong, P. R. China;
4. Department of Medical Imaging Center, State Key Laboratory of Oncology in South China, Guangdong Key Laboratory of Nasopharyngeal Carcinoma Diagnosis and Therapy, Guangdong Provincial Clinical Research Center for Cancer, Sun Yat-sen University Cancer Center, Guangzhou, Guangdong, P. R. China;
5. Department of Nasopharyngeal Carcinoma, State Key Laboratory of Oncology in South China, Guangdong Key Laboratory of Nasopharyngeal Carcinoma Diagnosis and Therapy, Guangdong Provincial Clinical Research Center for Cancer, Sun Yat-sen University Cancer Center, Guangzhou, Guangdong, P. R. China;
6. Department of Radiation Oncology, State Key Laboratory of Oncology in South China, Guangdong Key Laboratory of Nasopharyngeal Carcinoma Diagnosis and Therapy, Guangdong Provincial Clinical Research Center for Cancer, Sun Yat-sen University Cancer Center, Guangzhou, Guangdong, P. R. China.

^†^Chuanbo Xie, Long-Jun He, Wencheng Tan, Jindong Xie, and Yin Li contributed equally to this article.

^*^Corresponding authors:

Jian-Jun Li, Department of Endoscopy, Sun Yat-sen University Cancer Center, 651 Dongfeng Road East, Guangzhou, 510060, Guangdong, P. R. China; Email: lijj@sysucc.org.cn.

Jun Ma, Department of Radiation Oncology, Sun Yat-sen University Cancer Center, 651 Dongfeng Road East, Guangzhou, 510060, Guangdong, P. R. China; Email: majun@sysucc.org.cn.

Guokai Feng, State Key Laboratory of Oncology in South China, Sun Yat-sen University Cancer Center, 651 Dongfeng Road East, Guangzhou, 510060, Guangdong, P. R. China; Email: fengguok@sysucc.org.cn.

**Supplementary Materials and Methods**

**Participants**

From April 2016 to June 2020, 149 patients who were previously diagnosed with NPC (irrespective of previous treatments) and had enlarged RLNs on MRI after at least 6 months following the completion of radiotherapy at the Sun Yat-sen University Cancer Center (SYSUCC; Guangzhou, China) were screened and selected into this retrospective study. The inclusion criteria were: (1) age between 16-75 years; (2) absence of other malignancies; and (3) without disseminated disease on MRI images. The exclusion criteria were: (1) patients with nasal stenosis; (2) patients with coagulation dysfunction; (3) patients with distant metastasis; (4) patients with poorly controlled hypertension (5) RLNs that are lateral and posterior to the ICA; (6) patients with RLNs encasing the ICA. The initial TNM stage which was staged according to the 8th edition of the American Joint Committee on Cancer staging system, and was retrieved by reviewing medical records. Before the EPUS-TPNA examinations, all NPC patients were requested to sign an informed consent form. This study was approved by the Institutional Review Board of SYSUCC (approval number: XJS2016-016-01), and was registered at ClinicalTrials.gov (accession number: NCT03006588).

**MRI scan for the NPC patients**

All the selected participants had undergone cranial MRI image scanning with a 1.5-3.0T system (Signa, General Electric, CV/i; General Electric Healthcare, Chalfont St. Giles, United Kingdom). The scan region ranged from the suprasellar cistern to the inferior margin of the sternal end of the clavicle. T1-weighted images of the axial, coronal and sagittal planes (repetition time msec/echo time msec, 575/17) and T2-weighted images in the axial plane (2850/102) were obtained before injection of the contrast material. After intravenous injection with gadopentetate dimeglumine (Magnevist; Bayer Schering Pharma, Berlin, Germany) at a dose of 0.1 mmol per kilogram of body weight, T1-weighted fat-suppressed axial, coronal and sagittal sequences were sequentially performed with parameters like those used at imaging before injection of gadopentetate dimeglumine. The section thicknesses and intersection gaps were 5 mm and 1 mm for the axial plane, and 6 mm and 1 mm for the coronal and sagittal planes, respectively [1].

**Image assessment for the NPC patients**

Two radiologists separately evaluated the MRI images. Any disagreements were resolved by mutual discussions to reach a final agreement. RLN assessment included the medial and lateral LNs of the retropharyngeal space. The minimal and maximal axial diameters (perpendicular to the course of the internal jugular vein) and the longitudinal diameter of each visible RLN were measured on the MRI images. The minimal axial diameter corresponded to the widest diameter of the LN in the axial plane perpendicular to the maximal axial diameter. The longitudinal diameter paralleled the course of the internal jugular vein. Axial diameter measurements were made on T2-weighted axial images, and longitudinal diameter measurements were made on non-enhanced T1-weighted coronal images. MRI criteria for positive RLNs included: (1) the minimal RLN axial diameter with a minimal axial diameter of 6 mm or larger; (2) exhibited central necrosis;(3) the presence of multiple enlarged RLNs; and (4) medial RLNs.

**EPUS-TPNA procedure**

EPUS-TPNA procedure can be divided into: (1) Preoperative Preparation: Evaluate the patient's condition to select the appropriate puncture needle and determine the bronchoscope insertion route; Obtain a detailed patient history, including allergic reactions and bleeding tendencies; Prepare necessary emergency equipment and supplies, such as a resuscitation bag and hemostatic agents; Instruct the patient to fast and abstain from fluids for at least 4 hours prior to the procedure. (2) Anesthesia and Positioning: Ask the patient to lie in a supine position; Administer anesthetic agents and vasoconstrictors intranasally. (3) Bronchoscope Insertion: The operator holds the bronchoscope with the left hand and inserts it through the patient's oral or nasal cavity; Advance the endobronchial ultrasound bronchoscope to the site of the lesion. (4) Ultrasound Exploration: Sequentially examine lymph nodes or masses using ultrasound imaging; Conduct targeted scanning of the target lymph nodes and surrounding blood vessels in conjunction with imaging studies; Observe and measure the target lesion. (5) Ultrasound-Guided Puncture Biopsy: Use color Doppler flow imaging to avoid large blood vessels and identify the puncture site; Insert an EUS instrument (BF-UC 260F-OL8; Olympus, Tokyo, Japan) via the nostril and nasopharynx to scan the retropharyngeal space. Under real-time EUS guidance, a dedicated 22-gauge aspiration needle (NA-201SX-4022; Olympus) was used to puncture the enlarged RLN, and the needle was then withdrawn under 10 ml of suction pressure (Supplementary Video S1). Finally, the obtained strip-shaped tissue samples were fixed using the tissue fixative (CITOTEST) and subsequently sent for pathological and cytological examinations.

**Safety and efficacy assessment of EPUS-TPNA**

The assessment of safety was based on the occurrence of adverse events such as EPUS-TPNA-related bleeding, subcutaneous emphysema, choking, dyspnea, abnormal sensations, extremity paralysis, or hemiplegia during or following the EPUS-TPNA procedure. The evaluation of efficacy focused on the successful acquisition of tissues or cells from the RLNs via EPUS-FNA and the adequacy of these samples for subsequent pathological or cytological analysis [2].

**Ascertainment of RLNs recurrence**

All the patients in the EPUS-TPNA group received the first round EPUS-TPNA examination. After the first round EPUS-TPNA examinations, patients in the EPUS-TPNA group whose pathology showed the presence of cancer cells were confirmed as RLN recurrence. Patients whose RLNs were still cancer cell negative would undergo second or third round EPUS-TPNAs to further determine whether they were true non-NPC recurrence. Of note, if the third EPUS-TPNA results were still negative, the patients were closely followed up every 3 months by MRI until the end of the study or the day of death. The participants in the MRI-only group were followed up by MRI only from the first day of detecting enlarged RLN to either the day of death or the day of the last examination every 3 months during the first 2 years, every 6 months during the next 2 to 3 years, and annually thereafter until death. Patients in both the EPUS-TPNA group or MRI-only group with decreased or increased size of RLN after treatments were defined as NPC recurrence in RLN, while those with a stable size of RLN after treatments or during the follow-up period were defined as negative according to the Response Evaluation Criteria in Solid Tumors (RECIST version 1.1) [3].

**Statistical analysis**

Mean and standard deviation (SD) were used to describe continuous variables, and percentages to describe categorical variables. The clinical characteristics and treatment outcomes of the EPUS-TPNA group and MRI-only group were compared and analyzed using the chi-square test or t-test. Biopsy results and MRI follow-up outcomes were used as the golden standard to calculate the accuracy, sensitivity, specificity, positive predictive value (PPV), and negative predictive value (NPV) of EPUS-TPNA and MRI in diagnosing NPC recurrence in RLNs. We compared the treatment differences between the EPUS-TPNA group and the MRI-only group using the chi-square test. A two-tailed P value of < 0.05 was considered statistically significant. All the statistical analyses were performed by the R and SAS software.

**Supplementary Table S1. Characteristics of NPC patients with suspicious recurrent RLN.**

| **Characteristics** | **Diagnosis modality of enlarged RLN** | | ***P* value** |
| --- | --- | --- | --- |
|  | **EPUS-TPNA group**  **(72 cases)** | **MRI-only group**  **(77 cases)** |  |
| **Sex, *n* (%)** |  |  | 0.359 |
| Male | 57 (79.2) | 56 (72.7) |  |
| Female | 15 (20.8) | 21 (27.3) |  |
| **Age in years, mean ± SD** | 46.28 ± 10.45 | 46.17 ± 10.05 | 0.948 |
| **Age level, *n* (%)** |  |  | 0.695 |
| < 30 years | 2 (2.8) | 3 (3.9) |  |
| 30-60 years | 62 (86.1) | 68 (88.3) |  |
| > 60 years | 8 (11.1) | 6 (7.8) |  |
| **T stage of initial diagnosis by MRI, *n* (%)** |  |  | 0.563 |
| T1 | 4 (5.6) | 8 (10.4) |  |
| T2 | 10 (13.9) | 14 (18.2) |  |
| T3 | 47 (65.3) | 46 (59.7) |  |
| T4 | 11 (15.3) | 9 (11.7) |  |
| **N stage of initial diagnosis by MRI, *n* (%)** |  |  | 0.883 |
| N0 | 16 (22.2) | 15 (19.5) |  |
| N1 | 25 (34.7) | 24 (31.2) |  |
| N2 | 22 (30.6) | 28 (36.4) |  |
| N3 | 9 (12.5) | 10 (13.0) |  |
| **RLN detected in initial diagnosis by MRI, *n* (%)** |  |  | 0.246 |
| Present | 46 (63.9) | 56 (72.7) |  |
| Absent | 26 (36.1) | 21 (27.3) |  |
| **RLN detected by follow-up MRI^a^, *n* (%)** |  |  | 0.482 |
| Right lateral RLN only | 18 (25.0) | 16 (20.8) |  |
| Left lateral RLN only | 19 (26.4) | 16 (20.8) |  |
| Bilateral RLNs | 35 (48.6) | 45 (58.4) |  |
| **RLN size (minimal axial diameter), *n* (%)** |  |  | 0.261 |
| < 6 mm | 9 (12.5) | 4 (5.2) |  |
| 6-10 mm | 34 (47.2) | 47 (61.0) |  |
| > 10 mm | 29 (40.3) | 26 (33.8) |  |
| **RLN number, *n* (%)** |  |  | 0.492 |
| Single | 33 (45.8) | 31 (40.3) |  |
| Multiple | 39 (54.2) | 46 (59.7) |  |
| **RLN necrosis, *n* (%)** |  |  | 0.529 |
| Present | 16 (22.2) | 14 (18.2) |  |
| Absent | 56 (77.8) | 63 (81.8) |  |
| **Times of EPUS-TPNA, *n* (%)** |  |  | N/A |
| 1 time | 65 (90.3) | N/A |  |
| 2 times | 6 (8.3) | N/A |  |
| 3 times | 1 (1.4) | N/A |  |

**^a^**Refers to those confirmed by surgery, complete or partial remission (according to the RECIST 1.1 criteria) by radiotherapy or systemic therapy (i.e., chemotherapy, immunotherapy, or target therapy).

Abbreviations: EPUS-TPNA, endopharyngeal ultrasound-guided transpharyngeal needle aspiration; RLN, retropharyngeal lymph node; MRI, magnetic resonance imaging; NPC, nasopharyngeal carcinoma; N/A, not applicable; RECIST, response evaluation criteria in solid tumors.

**Supplementary Table S2. Accuracy of EPUS-TPNA and MRI in distinguishing malignant RLNs from benign RLNs after follow-up.**

| **Test result** | **Follow-up result** | |
| --- | --- | --- |
|  | **Positive RLNs^a^, cases** | **Negative RLNs^b^, cases** |
| **MRI-only group** |  |  |
| MRI-defined metastatic RLNs | 50 | 21 |
| MRI-defined non-metastatic RLNs | 4 | 2 |
| Total | 54 | 23 |
| **EPUS-TPNA group** |  |  |
| EPUS-TPNA-defined metastatic RLNs | 54 | 0 |
| EPUS-TPNA-defined non-metastatic RLNs | 1 | 17 |
| Total | 55 | 17 |

^a^Refers to those confirmed by surgery, complete or partial remission (according to the RECIST 1.1 criteria) by radiotherapy or systemic therapy (i.e., chemotherapy, immunotherapy, or target therapy).

^b^Refers to those confirmed by surgery, stable disease (according to the RECIST criteria) by radiotherapy or systemic therapy (i.e., chemotherapy, immunotherapy, or target therapy).

Abbreviations: EPUS-TPNA, endopharyngeal ultrasound-guided transpharyngeal needle aspiration; RLN, retropharyngeal lymph node; MRI, magnetic resonance imaging; RECIST, response evaluation criteria in solid tumors.

**Supplementary Video S1. Detailed operation video of EPUS-TPNA.**

[MP4]

**References**

1. Ma H, Liang S, Cui C, Zhang Y, Xie F, Zhou J, et al. Prognostic significance of quantitative metastatic lymph node burden on magnetic resonance imaging in nasopharyngeal carcinoma: A retrospective study of 1224 patients from two centers. Radiother Oncol. 2020;151:40-6.

2. He LJ, Xie C, Li Y, Luo LN, Pan K, Gao XY, et al. Ultrasound-guided fine needle aspiration of retropharyngeal lymph nodes after radiotherapy for nasopharyngeal carcinoma: a novel technique for accurate diagnosis. Cancer Commun (Lond). 2018;38(1):20.

3. Eisenhauer EA, Therasse P, Bogaerts J, Schwartz LH, Sargent D, Ford R, et al. New response evaluation criteria in solid tumours: revised RECIST guideline (version 1.1). Eur J Cancer. 2009;45(2):228-47.
